# Molecular cloning and expression analysis of *WRKY* transcription factor genes in *Salvia miltiorrhiza*

**DOI:** 10.1186/s12864-015-1411-x

**Published:** 2015-03-17

**Authors:** Caili Li, Dongqiao Li, Fenjuan Shao, Shanfa Lu

**Affiliations:** Institute of Medicinal Plant Development, Chinese Academy of Medical Sciences & Peking Union Medical College, No.151, Malianwa North Road, Haidian District, Beijing, 100193 China

## Abstract

**Background:**

WRKY proteins comprise a large family of transcription factors and play important regulatory roles in plant development and defense response. The *WRKY* gene family in *Salvia miltiorrhiza* has not been characterized.

**Results:**

A total of 61 *SmWRKYs* were cloned from *S. miltiorrhiza*. Multiple sequence alignment showed that SmWRKYs could be classified into 3 groups and 8 subgroups. Sequence features, the WRKY domain and other motifs of SmWRKYs are largely conserved with *Arabidopsis* AtWRKYs. Each group of WRKY domains contains characteristic conserved sequences, and group-specific motifs might attribute to functional divergence of WRKYs. A total of 17 pairs of orthologous *SmWRKY* and *AtWRKY* genes and 21 pairs of paralogous *SmWRKY* genes were identified. Maximum likelihood analysis showed that *SmWRKYs* had undergone strong selective pressure for adaptive evolution. Functional divergence analysis suggested that the *SmWRKY* subgroup genes and many paralogous *SmWRKY* gene pairs were divergent in functions. Various critical amino acids contributed to functional divergence among subgroups were detected. Of the 61 *SmWRKYs*, 22, 13, 4 and 1 were predominantly expressed in roots, stems, leaves, and flowers, respectively. The other 21 were mainly expressed in at least two tissues analyzed. In *S. miltiorrhiza* roots treated with MeJA, significant changes of gene expression were observed for 49 *SmWRKYs*, of which 26 were up-regulated, 18 were down-regulated, while the other 5 were either up-regulated or down-regulated at different time-points of treatment. Analysis of published RNA-seq data showed that 42 of the 61 identified SmWRKYs were yeast extract and Ag^+^-responsive. Through a systematic analysis, *SmWRKYs* potentially involved in tanshinone biosynthesis were predicted.

**Conclusion:**

These results provide insights into functional conservation and diversification of *SmWRKYs* and are useful information for further elucidating *SmWRKY* functions.

**Electronic supplementary material:**

The online version of this article (doi:10.1186/s12864-015-1411-x) contains supplementary material, which is available to authorized users.

## Background

*Salvia miltiorrhiza* Bunge (Lamiaceae), known as danshen in Chinese, is one of the most important Traditional Chinese Medicine (TCM) materials. It has been widely used in Chinese medicines treating coronary heart disease, hepatitis, menstrual disorders, menostasis, blood circulation diseases, and other cardiovascular diseases [1]. The main bioactive components of *S. miltiorrhiza* include the water-soluble (hydrophilic) phenolics, such as rosmarinic acid, salvianolic acid A, salvianolic acid B and lithospermic acid [2], and the lipid-soluble (nonpolar, lipophilic) diterpenoids, known as tanshinones [3]. Enzymes catalyzing the biosynthesis of these bioactive components have been intensively studied recently [4-10]. A large number of genes involved in the biosynthesis of phenolics and terpenoids have been identified through either molecular cloning or transcriptome-wide characterization [3,11-17]. Collectively, *S. miltiorrhiza* is being developed to be a medicinal model plant [18].

Transcription factors are a class of significant regulators controlling plant growth and development through regulating gene expression at the transcriptional level. They bind to the specific regions, known as *cis*-elements, in the promoters of genes and then activate or repress the expression of regulated genes in collaboration with other regulatory factors. So far, two large transcription factor gene families, including the plant-specific SQUAMOSA promoter-binding protein-like (*SPL*) transcription factor gene family and the largest plant transcription factor gene family, termed *MYB*, have been characterized in *S. miltiorrhiza* [19,20]. A total of 15 *SmSPLs* and 110 *SmMYBs* have been identified from *S. miltiorrhiza. SmSPLs* are involved in the regulation of developmental timing in *S. miltiorrhiza* and eight of them are targets of miR156/157 [19]. Similarly, a subset of *SmMYBs* is regulated by microRNAs, such as miR159, miR319, miR828 and miR858. Many *SmMYBs* are involved in the biosynthesis of bioactive compounds in *S. miltiorrhiza* [20].

*WRKY* is a large transcription factor gene family specific to the green lineage, including green algae and land plants. The first *WRKY* gene, known as *SPF1*, was cloned from *Ipomoea batatas* about twenty year ago [21]. Since then, great progress has been achieved in *WRKY* gene identification and functional analysis. Plants with *WRKYs* identified include green alga (*Chlamydomonas reinhardtii*) [22], moss (*Physcomitrella patens*) [22], fern (*Ceratopteris richardii*) [22], pine (*Pinus monticola*) [23], *Arabidopsis* [24], tobacco (*Nicotiana tabacum*) [25-27], rice (*Oryza sativa*) [28,29], soybean (*Glycine max*) [30], maize (*Zea mays*) [31], barley (*Hordeum vulgare*) [32], grape (*Vitis vinifera*) [33,34], poplar (*Populus trichocarpa*) [35], tomato (*Solanum lycopersicum*) [36], cucumber (*Cucumis sativus*) [37], coffee (*Coffea arabica*) [38], and so forth.

Characterization of the identified *WRKY* genes showed that they were significant regulators involved in plant developmental processes and responsed to biotic and abiotic stresses [39]. The involvement of *WRKYs* in plant immune response against bacterial, fungal, and viral pathogens has been widely reported [40-51]. Recently, more and more evidence showed the regulatory roles of *WRKY* in plant response to abiotic stresses. For example, over-expression of three soybean *WRKY* genes (*GmWRKY13*, *GmWRKY27* and *GmWRKY54*) in *Arabidopsis* showed that *GmWRKY21*-transgenic *Arabidopsis* plants were tolerant to cold stress, *GmWRKY54* conferred salt and drought tolerance, whereas transgenic plants over-expressing *GmWRKY13* had increased sensitivity to salt and mannitol stresses and decreased sensitivity to abscisic acid [52]. It suggests the involvement of *WRKY* genes in multiple abiotic stress-associated signaling pathways and the association of different *WRKY* members with different abiotic stresses. Moreover, *WRKYs* associated with same abiotic stress may show different responses. For instance, *Arabidopsis WRKY25* and *WRKY26* are heat-induced, whereas *WRKY33* is heat-repressed [53]. In addition to stress responses, *WRKYs* also regulate various developmental processes, such as seed dormancy and germination, flowing, fruit maturation, stem elongation, pith secondary cell wall formation, plant senescence, and trichome development [54-58]. It suggests the importance of *WRKYs* and the complexity of *WRKY*-associated regulatory networks.

The defining feature of WRKY transcription factors is their DNA-binding domain, known as WRKY domain [39]. It is approximately 60 amino acids in length and includes the conserved amino acid sequence WRKYGQK at the N-terminus and an atypical zinc-finger motif either C2H2 (C–X_4–5_–C–X_22–23_–H–X_1_–H) or C2HC (C–X_7_–C–X_23_–H–X_1_–C) at the C-terminus. The structure of the WRKY domain allows it to specifically interact with W-box and SURE (sugar responsive) *cis*-elements in the promoter of target genes [59-61]. WRKY can be divided into three groups (Groups I, II and III) based on the number of WRKY domains (two domains in Group I and one in the others) and the pattern of zinc finger motif (C2H2 in Groups I and II and C2HC in Group III) [39,40]. Additionally, Group II WRKY proteins can be further divided into subgroups, including IIa, IIb, IIc, IId and IIe, based on the primary amino acid sequence of the WRKY domain.

Although *WRKYs* have been identified and characterized in various plant species, no information is available for the *WRKY* gene family in *S. miltiorrhiza*. In this study, we cloned and characterized 61 *S. miltiorrhiza SmWRKYs*.

## Results and discussion

### Molecular cloning of 61 *SmWRKY* genes from *S. miltiorrhiza*

It has been shown that 72 *AtWRKY* genes exist in the *Arabidopsis* genome (Additional file 1: Table S1). To identify *SmWRKYs*, BLAST analysis against the current assembly of the *S. miltiorrhiza* genome was performed using AtWRKY protein sequences as queries. A total of 61 gene models were predicted for *SmWRKYs*. The 5’-sequence of many *SmWRKYs* showed low homology with known plant *WRKYs*. It might cause errors in computational prediction. To verify the predicted gene models and correct errors of computation, full-length coding sequences (CDSs) of all 61 *SmWRKYs* were PCR-amplified using the primers listed in Additional file 2: Table S2 and then cloned and sequenced. It resulted in the identification of 61 *SmWRKYs*, which were named *SmWRKY1*–*SmWRKY61*, respectively. The deduced SmWRKY proteins have amino acid numbers from 129 to 706, isoelectric points (p*I*) from 4.76 to 9.9, and molecular weights (Mw) from 19.9 to 76.2 kDa. All of the 61 cloned CDSs have been submitted to GenBank under the accession numbers shown in Table 1. The number of identified *SmWRKYs* is comparable with that in *Arabidopsis*. Comparable gene numbers were also found for the *MYB* [20], *SPL* [19], *Argonaute* (*AGO*) [62] and *RNA-dependent RNA polymerase* (*RDR*) [63] gene families in *S. miltiorrhiza* and *Arabidopsis*. It suggests that *S. miltiorrhiza* and *Arabidopsis* may have similar number tfmkof gene members for many gene families. Thus, the identified *SmWRKYs* represent an almost complete set of *WRKYs* in *S. miltiorrhiza*, although it may be not a fully complete set.

**Table 1:** **Sequence features of WRKYs in** ***S. miltiorrhiza***

**Name**	**Gene ID**	**AA Len**	**pI**	**Mw (Da)**	**Group**	**Conserved motif**	**Domain pattern**	**Zinc finger**
SmWRKY1	KM823124	486	5.79	52075.53	2b	WRKYGQK	C-X5-C-X23-HXH	C2H2
SmWRKY2	KM823125	526	7.2	57916.44	1	2×[WRKYGQK]	C-X4-C-X22-HXH	C2H2
SmWRKY3	KM823126	295	9.84	31785.84	2d	WRKYGQK	C-X5-C-X23-HXH	C2H2
SmWRKY4	KM823127	292	7.05	32163.19	2c	WRKYGQK	C-X4-C-X23-HXH	C2H2
SmWRKY5	KM823128	283	6.6	31674.21	2e	WRKYGQK	C-X5-C-X23-HXH	C2H2
SmWRKY6	KM823129	332	9.6	36424.23	2d	WRKYGQK	C-X5-C-X23-HXH	C2H2
SmWRKY7	KM823130	262	6.18	29689.04	3	WRKYGQK	C-X7-C-X23-HXC	C2HC
SmWRKY8	KM823131	300	5.18	33675.01	2e	WRKYGQK	C-X5-C-X23-HXH	C2H2
SmWRKY9	KM823132	269	8.91	29956.66	2a	WRKYGQK	C-X5-C-X23-HNH	C2H2
SmWRKY10	KM823133	343	5.37	38240.49	3	WRKYGQK	C-X7-C-X23-HXC	C2HC
SmWRKY11	KM823134	349	5.34	39120.3	3	WRKYGQK	C-X7-C-X23-HXC	C2HC
SmWRKY12	KM823135	211	8.11	24263.86	2c	WRKYGQK	C-X4-C-X23-HXH	C2H2
SmWRKY13	KM823136	435	5.7	47512.82	1	2×[WRKYGQK]	C-X4-C-X22-HXH	C2H2
SmWRKY14	KM823137	243	8.19	27565.82	2c	WRKYGKK	C-X4-C-X23-HXH	C2H2
SmWRKY15	KM823138	549	6.39	59091.35	2b	WRKYGQK	C-X5-C-X23-HXH	C2H2
SmWRKY16	KM823139	321	5.02	35934.8	3	WRKYGQK	C-X7-C-X23-HXC	C2HC
SmWRKY17	KM823140	157	9.46	18126.30	2c	WRKYGQK	C-X4-C-X23-HXH	C2H2
SmWRKY18	KM823141	333	4.76	36599.98	2e	WRKYGQK	C-X5-C-X23-HXH	C2H2
SmWRKY19	KM823142	221	9	25422.86	2c	WRKYGQK	C-X4-C-X23-HXH	C2H2
SmWRKY20	KM823143	328	9.56	35953	2d	WRKYGQK	C-X5-C-X23-HXH	C2H2
SmWRKY21	KM823144	309	6.18	34870.74	2c	WRKYGQK	C-X4-C-X23-HXH	C2H2
SmWRKY22	KM823145	407	8.72	44553.66	2b	WRKYGQK	C-X5-C-X23-HXH	C2H2
SmWRKY23	KM823146	341	9.61	37956.92	2d	WRKYGQK	C-X5-C-X23-HXH	C2H2
SmWRKY24	KM823147	364	7.66	40559.08	1	2×[WRKYGQK]	C-X4-C-X22-HXH	C2H2
SmWRKY25	KM823148	329	5.9	36268.73	2c	WRKYGQK	C-X4-C-X23-HXH	C2H2
SmWRKY26	KM823149	445	6.33	49392.18	2b	WRKYGQK	C-X5-C-X23-HXH	C2H2
SmWRKY27	KM823150	346	9.77	37310.40	2d	WRKYGQK	C-X5-C-X23-HXH	C2H2
SmWRKY28	KM823151	486	8.38	53222.94	--	2×[WRKYGQK]	C-X4-C-X22-HXH	C2H2
SmWRKY29	KM823152	519	6.13	63235.29	2b	WRKYGQK	C-X5-C-X23-HXH	C2H2
SmWRKY30	KM823153	509	6.84	55355.55	2b	WRKYGQK	C-X5-C-X23-HXH	C2H2
SmWRKY31	KM823154	516	8.6	55885.41	1	2×[WRKYGQK]	C-X_4_-C-X_22_-HXH	C2H2
SmWRKY32	KM823155	129	9.3	15217.26	2c	WRKYGQK	C-X_4_-C-X_23_-HXH	C2H2
SmWRKY33	KM823156	175	9.36	19944.38	2c	WRKYGQK	C-X_4_-C-X_23_-HXH	C2H2
SmWRKY34	KM823157	309	8.11	34006.08	2a	WRKYGQK	C-X_5_-C-X_23_-HNH	C2H2
SmWRKY35	KM823158	508	6.12	55526.63	2b	WRKYGQK	C-X_5_-C-X_23_-HXH	C2H2
SmWRKY36	KM823159	246	6.61	27210.05	2c	WRKYGQK	C-X_4_-C-X_23_-HXH	C2H2
SmWRKY37	KM823160	290	6.6	32766.16	2c	WRKYGQK	C-X_4_-C-X_23_-HXH	C2H2
SmWRKY38	KM823161	284	9.41	30581.63	2d	WRKYGQK	C-X_5_-C-X_23_-HXH	C2H2
SmWRKY39	KM823162	390	6.64	43510.55	1	2×[WRKYGQK]	C-X_4_-C-X_22_-HXH	C2H2
SmWRKY40	KM823163	352	6.07	38849.51	2e	WRKYGQK	C-X_5_-C-X_23_-HXH	C2H2
SmWRKY41	KM823164	587	9.12	64687.26	1	2×[WRKYGQK]	C-X_4_-C-X_22_-HXH	C2H2
SmWRKY42	KM823165	706	6.02	76197.45	1	2×[WRKYGQK]	C-X_4_-C-X_22_-HXH	C2H2
SmWRKY43	KM823166	179	5.39	19963.09	2c	WRKYGKK	C-X_4_-C-X_23_-HXH	C2H2
SmWRKY44	KM823167	265	5.73	28206.05	2c	WRKYGQK	C-X_4_-C-X_23_-HXH	C2H2
SmWRKY45	KM823168	336	5.95	37211.08	3	WRKYGQK	C-X_7_-C-X_23_-HXC	C2HC
SmWRKY46	KM823169	306	6.41	33426.96	2e	WRKYGQK	C-X_5_-C-X_23_-HXH	C2H2
SmWRKY47	KM823170	268	5.86	30225.72	2c	WRKYGQK	C-X_4_-C-X_23_-HXH	C2H2
SmWRKY48	KM823171	224	5.88	25174.62	2e	WRKYGQK	C-X_5_-C-X_23_-HXH	C2H2
SmWRKY49	KM823172	351	9.9	39451.59	2d	WRKYGQK	C-X_5_-C-X_23_-HXH	C2H2
SmWRKY50	KM823173	291	5.45	33184.06	2e	WRKYGQK	C-X_5_-C-X_23_-HXH	C2H2
SmWRKY51	KM823174	526	7.2	57916.44	1	2×[WRKYGQK]	C-X_4_-C-X_22_-HXH	C2H2
SmWRKY52	KM823175	171	6.66	19066.92	2c	WRKYGQK	C-X_4_-C-X_23_-HXH	C2H2
SmWRKY53	KM823176	575	6.54	62764.4	1	2×[WRKYGQK]	C-X_4_-C-X_22_-HXH	C2H2
SmWRKY54	KM823177	491	7.69	53504.9	1	2×[WRKYGQK]	C-X_4_-C-X_22_-HXH	C2H2
SmWRKY55	KM823178	449	9.12	49222.93	1	2×[WRKYGQK]	C-X_4_-C-X_22_-HXH	C2H2
SmWRKY56	KM823179	297	5.34	33782.19	2e	WRKYGQK	C-X_5_-C-X_23_-HXH	C2H2
SmWRKY57	KM823180	281	5.18	31675.83	2c	WRKYGQK	C-X_4_-C-X_23_-HXH	C2H2
SmWRKY58	KM823181	272	6.32	30091.63	2a	WRKYGQK	C-X_5_-C-X_23_-HNH	C2H2
SmWRKY59	KM823182	352	8.34	38285.97	2b	WRKYGQK	C-X_5_-C-X_23_-HXH	C2H2
SmWRKY60	KM823183	379	7.63	42242.82	1	2×[WRKYGQK]	C-X_4_-C-X_22_-HXH	C2H2
SmWRKY61	KM823184	168	8.96	19153.70	3	WRKYGQK	C-X_7_-C-X_23_-HXC	C2HC

### Classification of the WRKY domains and the WRKY proteins

**Figure 1:**
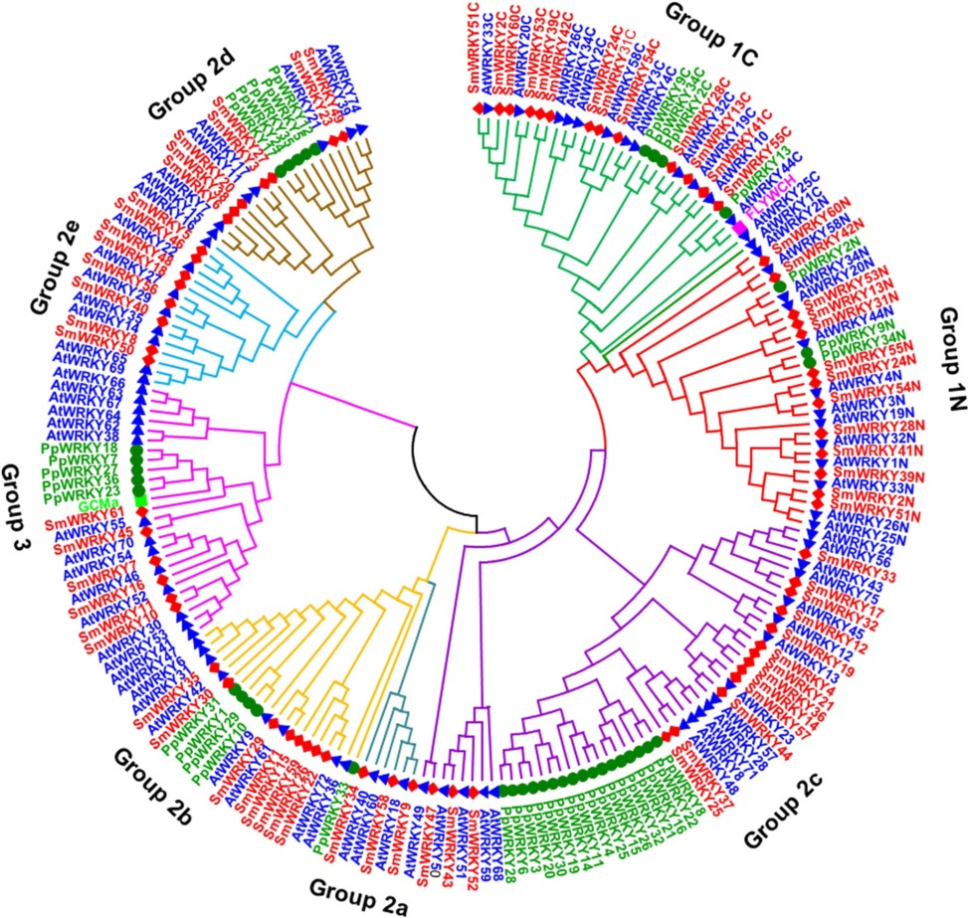
**Phylogenetic tree of the WRKY domain from SmWRKYs (red), AtWRKYs (blue). PpWRKYs (dark green), FLYWCH (pink) and GCMa (light green).** Groups/subgroups are shown; ‘N’ and ‘C’ indicate the N-terminal and C-terminal WRKY domain of a specific WRKY protein.

**Figure 2:**
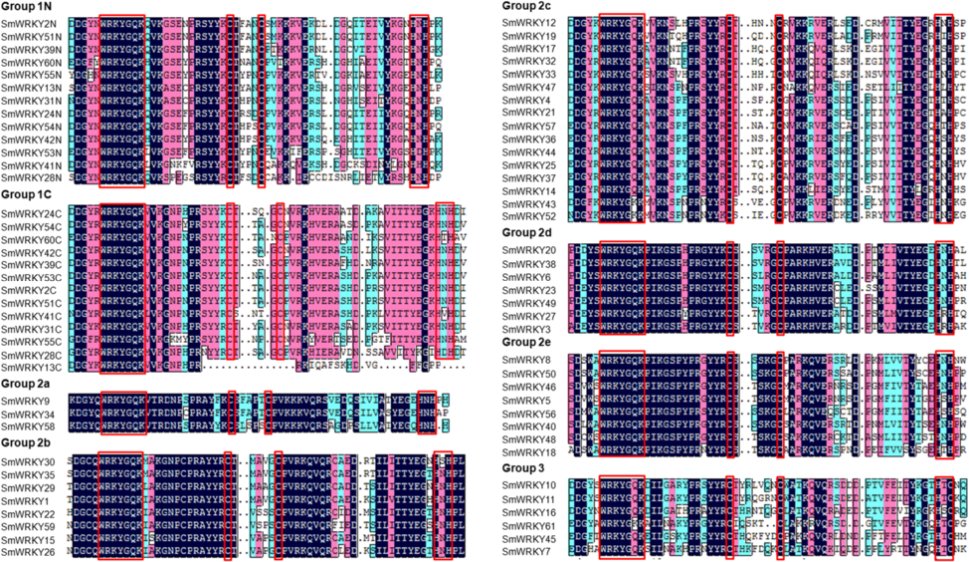
**Multiple sequence alignment of the WRKY domain from SmWRKYs.** Red box indicates conserved WRKY amino acid signature and zinc-finger motif; Black box indicates conserved amino acids. ‘N’ and ‘C’ indicate the N-terminal and C-terminal WRKY domain of a specific WRKY protein.

**Figure 3:**
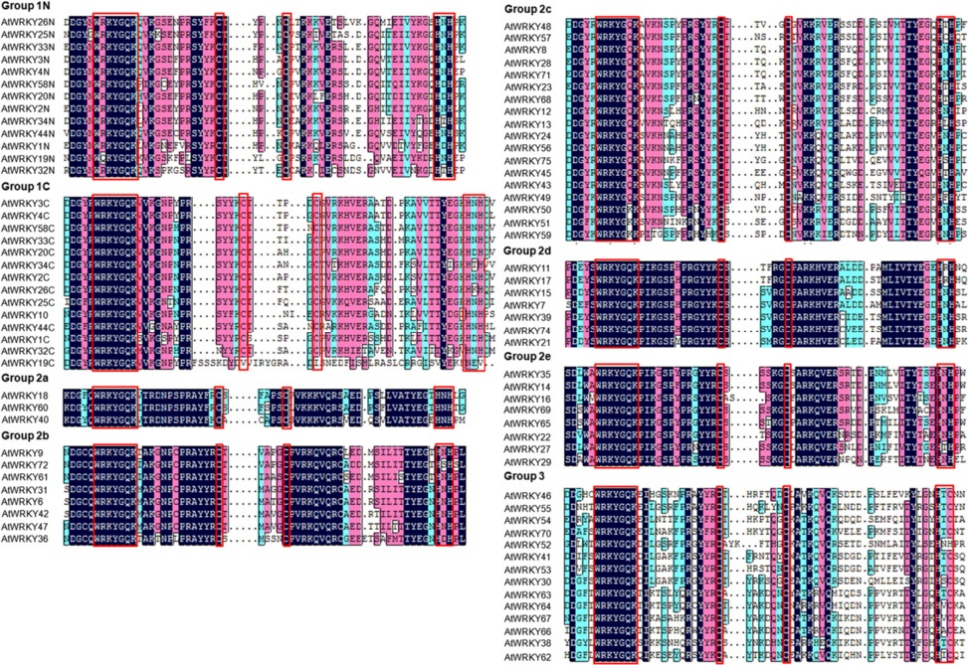
**Multiple sequence alignment of the WRKY domain from AtWRKYs.** Red box indicates conserved WRKY amino acid signature and zinc-finger motif; Black box indicates conserved amino acids. ‘N’ and ‘C’ indicate the N-terminal and C-terminal WRKY domain of a specific WRKY protein.

Transcription factors in a family usually share highly conserved DNA-binding domains. In order to examine the phylogenetic relationships among WRKYs, a neighbor-joining (NJ) phylogenetic tree was constructed for the WRKY domain sequences of AtWRKYs, SmWRKYs, PpWRKYs, GCMa and FLYWCH using MEGA5.0 (Figure 1). According to the classification of AtWRKYs, the WRKY domains were divided into 3 groups (groups 1, 2 and 3). Group 1 WRKY domains come from proteins containing two WRKY domains, one of which is located in the N-terminal (NTWD), while the other one is in the C-terminal (CTWD). The exceptions within this group are the domains from AtWRKY10 and PpWRKY13, each of which possesses a single WRKY domain. Group 1 WRKY domains were further divided into two subgroups, termed group 1 N and group 1C, respectively. Based on their characteristics in the phylogenetic tree, group 2 WRKY domains could be classified into 5 subgroups, including groups 2a, 2b, 2c, 2d and 2e. Multiple sequence alignment of the core WRKY domains, each of which contains approximately 60 residues, showed that 71 of the 74 WRKY domains from 61 SmWRKYs contained the highly conserved sequence WRKYGQK, while the other three (SmWRKY43, SmWRKY52, and SmWRKY61) had WRKYGKK (Figure 2). Of the 61 SmWRKY, 13 are two-WRKY-domain-containing proteins and all of them have the C2H2-type zinc-finger motif (C–X_4_–C–X_22–23_–H–X_1_–H) (Figure 2, Table 2). The other 48 SmWRKY proteins are one-WRKY-domain-containing proteins, 42 of which contain group 2 WRKY domains and have the same type of finger motif (C–X_4–5_–C_23–24_–H–X_1_–H), while the other 6 contain group 3 WRKY domains and have the C2HC zinc finger motif (C–X_7_–C_23_–H–X_1_–C) (Figure 2, Table 2). The distribution of residues in the WRKY domains of *S. miltiorrhiza* WRKY proteins is quite similar to that of *Arabidopsis* (Figures 2 and 3), suggesting evolutionary conservation of SmWRKYs and AtWRKYs. Comparing the number of SmWRKYs and AtWRKYs in each group/subgroup showed that the number of SmWRKYs in groups 1 and 2 is similar to that of AtWRKYs in the same group; however the number 6 of SmWRKY members belonging to group 3 is significantly less than the number 14 of AtWRKY members included in the same group. It is consistent with previous results showing group 3 WRKYs to be a newly defined and most dynamic group [22] and suggests the divergence of WRKYs in *S. miltiorrhiza* and *Arabidopsis*.

**Table 2:** **Number of WRKY domains from** ***S. miltiorrhiza*** **and** ***Arabidopsis***

**Group**	**Subgroup**	**Gene number**				**Consensus**	**Exception**
		**AtWRKY**		**SmWRKY**			
1	1 N	27	13	26	13	C-X_4_-C-X_22_-HXH	n = 23, AtWRKY26
	1C		14		13	C-X_4_-C-X_23_-HXH	
2	2a	44	3	42	3	C-X_5_-C-X_23_-HNH	
	2b		8		8	C-X_5_-C-X_23_-HXH	n = 24, AtWRKY36
	2c		18		16	C-X_4_-C-X_23_-HXH	
	2d		7		7	C-X_5_-C-X_23_-HXH	
	2e		8		8	C-X_5_-C-X_23_-HXH	
3		14	14	6	6	C-X_7_-C-X_23_-HXC	n = 22, m = 5, SmWRKY61
							AtWRKY52: HNH for HXC
Total		85		74

**Figure 4:**
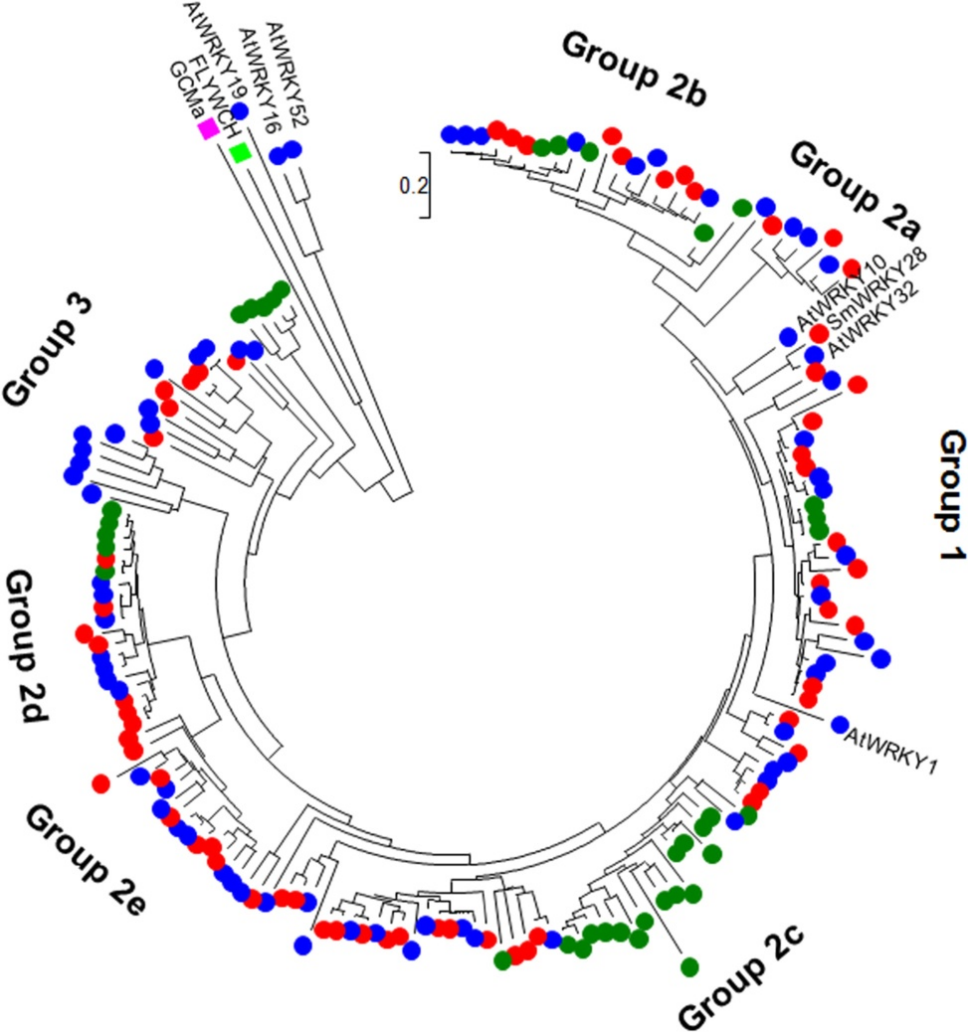
**Phylogenetic analysis of SmWRKYs (red), AtWRKYs (blue).** PpWRKYs (dark green), FLYWCH (light green) and GCMa (pink) proteins. The unrooted phylogenetic tree was constructed using the neighbor-joining method. The names of WRKY proteins not included in a group are shown.

In order to investigate whether the phylogenies are different between the WRKY domains and the corresponding WRKY proteins, we constructed an NJ tree based on the full-length amino acid sequences of SmWRKYs, AtWRKYs, PpWRKYs, GCMa and FLYWCH (Figure 4). The results showed that the phylogenetic tree of WRKY proteins was quite similar to the tree of WRKY domains with little difference observed (Figures 1 and 4). For instance, AtWRKY1, AtWRKY32 and SmWRKY28 having two WRKY domains and AtWRKY10 belonging to group 1 WRKY domains form separated clades outside group 1. AtWRKY19 and FLYWCH with the WRKY domain belonging to group 1, AtWRKY16 with the WRKY domain belonging to group 2e, and AtWRKY52 and GCMa with the WRKY domain belonging to group 3 form separated clades outside groups 1, 2 and 3. These results indicate the difference between the WRKY domain and the sequence outside the WRKY domain. Based on the NJ tree constructed with full-length amino acid sequences, we identified 17 pairs of orthologous *WRKY* genes in *S. miltiorrhiza* and *Arabidopsis* (Table 3). It suggests that many *SmWRKY*s and *AtWRKYs* are evolutionarily conserved.

**Table 3:** **WRKY orthologs in** ***S. miltiorrhiza*** **and** ***Arabidopsis***

***SmWRKYs***	**Putative** ***Arabidopsis*** **orthologs**	**Phylogenetic group in the NJ tree**
*SmWRKY55*	*AtWRKY44*	Group 1
*SmWRKY54*	*AtWRKY3/AtWRKY4*	Group 1
*SmWRKY53*	*AtWRKY20*	Group 1
*SmWRKY51/SmWRKY2*	*AtWRKY33*	Group 1
*SmWRKY28*	*AtWRKY32*	Out of group 1
*SmWRKY34/SmWRKY58*	*AtWRKY40*	Group 2a
*SmWRKY59*	*AtWRKY72*	Group 2b
*SmWRKY57*	*AtWRKY23*	Group 2c
*SmWRKY12*	*AtWRKY12*	Group 2c
*SmWRKY19*	*AtWRKY13*	Group 2c
*SmWRKY47*	*AtWRKY49*	Group 2c
*SmWRKY49*	*AtWRKY39*	Group 2d
*SmWRKY56*	*AtWRKY29*	Group 2e
*SmWRKY40*	*AtWRKY35/AtWRKY14*	Group 2e
*SmWRKY8*	*AtWRKY27*	Group 2e
*SmWRKY45*	*AtWRKY55*	Group 3
*SmWRKY16*	*AtWRKY30*	Group 3

### Analysis of conserved motifs

**Figure 5:**
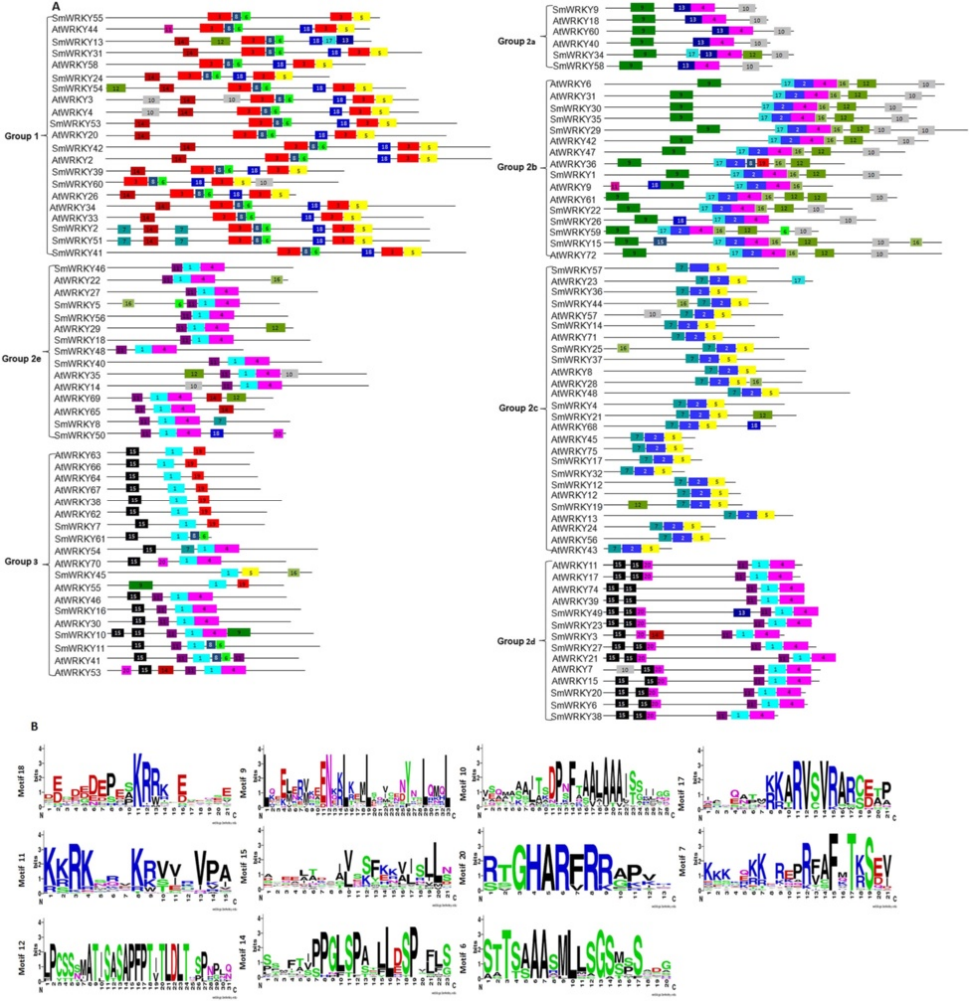
**Architecture of conserved protein motifs in SmWRKYs and AtWRKYs. A**: Architecture of conserved protein motifs in SmWRKYs and AtWRKYs from different groups (or subgroups). Motifs represented with boxes are predicted using MEME. The number in boxes (1–20) represents motif 1–motif 20, respectively. Box size indicates the length of motifs; **B**: Sequence logo of eleven conserved motifs, including motif 7, motif 9–motif 12, motif 14–motif 18 and motif 20. The logos were created on the WebLogo server (http://weblogo.berkeley.edu/logo.cgi). Bits represent the conservation of sequence at a position.

In addition to the WRKY domain, other conserved motifs could be important for the diversified functions of WRKY proteins from *S. miltiorrhiza* and *Arabidopsis* [64,65]. Using the MEME program, we identified a total of 20 conserved motifs in WRKYs from *S. miltiorrhiza* and *Arabidopsis* (Figure 5). The length of motifs varies from 8 to 150 amino acids and the number of motifs in each WRKY varies between 2 and 11. The majority of the identified motifs were found in more than one subgroup of WRKYs. Many AtWRKYs in a subgroup contain the same motif(s) as their SmWRKYs orthologues in the subgroup. It suggests the conservation of motifs in *S. miltiorrhiza* and *Arabidoopsis* WRKYs belonging to a subgroup.

Among the 20 conserved motifs, 9 motifs, including motifs 1, 2, 3, 4, 5, 6, 8, 13, and 19, are located in the WRKY domain, while the other 11 are located outside the domain. Most WRKY proteins in a group have similar motif compositions (Figure 5). For instance, motif 18 exists in almost all of group 1 WRKY proteins except for SmWRKY55. Motif 14 exists in 16 of the 21 group 1 *S. miltiorrhiza* and *Arabidopsis* WRKY proteins. Motifs 9 and 10 commonly exist in groups 2a and 2b, two close subgroups in the phylogenetic tree. Motifs 12, 16 and 17 exist in most WRKY proteins of group 2b but only in a few members of 2a. Motif 7 exists in all of the WRKY proteins belonging to group 2c. Motif 11 is shared by proteins belonging to groups 2d and 2e. Additionally, motif 15, known as the Ca^2+^-dependent CaM-binding domain (CaMBD) [66], commonly exists in most WRKY proteins belonging to groups 2d and 3. Group 2d WRKY proteins usually contain two motif 15 s, while the majority of group 3 proteins contain only one. Motif 20, known as the HARF motif [40,67], exists in 5 of 7 AtWRKYs and all 7 SmWRKYs in group 2d. The results indicate functional similarities of WRKY proteins belonging to a group. Group-specific motifs may attribute to functional divergence of WRKYs.

### Selective constraints on *SmWRKY* genes

In order to preliminarily examine the evolutionary mechanism of *WRKYs*, we test the hypothesis of positive selection acting on *SmWRKY* genes using site models and branch-site models in PAML [68] developed by Nielsen and Yang [69] and Yang et al. [70]. Codon substitution models [71] M0, M1a, M2a, M3, M7 and M8 were applied to the alignments and these models assume variation in ω among sites. The parameter estimates, log-likelihood and the LRT tests for these models are shown in Table 4. To examine how dN/dS ratios differed among codon positions, we compared models M0 and M3. The log likelihood of M0 for SmWRKY sequences was l = −5119.032, with an estimate of ω = 0.038. The low ω value suggests a strong action of purifying selection in the evolution of *SmWRKY* analyzed. M3 (discrete) assumes a general discrete distribution with three site classes (*p*_0_, *p*_1_, *p*_2_). The log likelihood of M3 was l = −4864.540, with an estimate of ω_0_ = 0.00052, ω_1_ = 0.02439, ω_2_ = 0.08755 (Table 4). Consistent with M0, the data from M3 also suggest that all codons are under purifying selection. Additionally, the value of twice the log likelihood difference (2ΔlnL) between M3 and M0 is 508.98. It is strongly statistically significant (p < 0.01) and suggests the overall level of selective constraints fluctuated.

**Table 4:** **Tests for positive selection among codons of** ***WRKY*** **genes using site models**

**Model**	**lnL**	**Estimates of parameter** ^**1**^		**2ΔlnL**	**Positive selection sites** ^**2**^
		**Frequency**	**dN/dS**		
M0(one-ratio)	−5119.032	p = 1.000	ω = 0.03756	508.984 (M3 vs. M0)**	Not allowed
M3(discrete)	−4864.540	p0 = 0.30493	ω0 = 0.00052		None
		p1 = 0.32215	ω1 = 0.02439		
		p2 = 0.37292	ω2 = 0.08755		
M1a(nearly neutral)	−5119.251	p0 = 0.93054	ω0 = 0.04576	0 (M2a vs. M1a)	Not allowed
		p1 = 0.06946	ω1 = 1.00000		
M2a(positive selection)	−5119.251	p0 = 0.93052	ω0 = 0.04576		None
		p1 = 0.03470	ω1 = 1.00000		
		p2 = 0.03478	ω2 = 1.00000		
M7(beta)	−4857.207	p = 0.39586		0.002 (M8 vs. M7)	Not allowed
		q = 9.86708			
M8(beta & ω)	−4857.206	p0 = 0.99999	ω = 1.35981		None
		p = 0.39661			
		p1 = 0.00001			
		q = 9.86579			

Note: *p < 0.05 and **p < 0.01 (*x* ^2^ test).

^1^ω was estimated under model M0,M3,M7, and M8; p and q are the parameters of the beta distribution.

^2^The number of amino acid sites estimated to have undergone positive selection.

To test whether positive selection promoted divergence between genes, the codon substitution models that allow positive selection (M2a and M8) and that hypothesize nearly neutral selection (M1a and M7) were compared (M2a vs. M1a and M8 vs. M7; Table 4). The log likelihood of M1a and M2a for *SmWRKY* sequences was l = −5119.251. However, no site was positively selected at a level of 95%. M7 and M8 fitted the sequences better than M0, M3, M1a and M2a with values of l = −4857.207 and −4857.206, respectively (Table 4). In both cases, no significant evidence of positive selection was found.

Branch-site models aim to detect positive selection affecting a few sites along particular lineages and allow ω ratios to vary among sites and lineages simultaneously [68]. It seems that the branch-site models are most suitable for describing evolutionary processes of the *WRKY* gene family. Therefore, we analyzed positively selected amino acid sites of SmWRKYs using the improved branch-site model [72]. The branches being tested for positive selection were used as the foreground, while all other branches on the tree were used as the background. The Bayes empirical Bayes (BEB) method was used to calculate the posterior probabilities. A codon is probably from the site class of positive selection if LRT suggested the presence of codons under positive selection on the foreground branch [73]. The parameter estimates for lineages under positive selection are given in Table 5. A total of 19 residues were found to be under positive selection (p > 90%). It includes 6 in group 2c and 10 in group 2d. The other three residues were found in group 2b, group 2e and group 3. No residues in group 1 and group 2a were found to be under positive selection. The results suggest that different WRKY groups may have different evolutionary rates. Groups 2c and 2d could be confronted with strong positive Darwinian selection, since many highly significant positive sites were detected at the 0.01 significance level (Table 5). The evolution in the other groups seems to be more conservative.

**Table 5:** **Parameters estimation and likelihood ratio tests for the branch-site models**

**Branch-site model**					
**Foreground branches**	**Estimates of parameter**				**Positive delection sites(BEB)**
	**Site class 0**	**Site class 1**	**Site class 2a**	**Site class 2b**	
	P_0_ = 0.10272	P_1_ = 0.36090	P_2a_ = 0.11884	P_2b_ = 0.41754	
Group 1	ω_0(b)_ = 0.05880	ω_1(b)_ = 1.00000	ω_2a(b)_ = 0.05880	ω_2b(b)_ = 1.00000	None
	ω_0(f)_ = 0.05880	ω_1(f)_ = 1.00000	ω_2a(f)_ = 1.00000	ω_2b(f)_ = 1.00000	
	P_0_ = 0.45646	P_1_ = 0.17985	P_2a_ = 0.26089	P_2b_ = 0.10279	
Group 2a	ω_0(b)_ = 0.05165	ω_1(b)_ = 1.00000	ω_2a(b)_ = 0.05165	ω_2b(b)_ = 1.00000	None
	ω_0(f)_ = 0.05165	ω_1(f)_ = 1.00000	ω_2a(f)_ = 1.00000	ω_2b(f)_ = 1.00000	
	P_0_ = 0.42991	P_1_ = 0.34434	P_2a_ = 0.12535	P_2b_ = 0.10040	
Group 2b	ω_0(b)_ = 0.11089	ω_1(b)_ = 1.00000	ω_2a(b)_ = 0.11089	ω_2b(b)_ = 1.00000	359 G*
	ω_0(f)_ = 0.11089	ω_1(f)_ = 1.00000	ω_2a(f)_ = 999.00000	ω_2b(f)_ = 999.00000	
	P_0_ = 0.20480	P_1_ = 0.56991	P_2a_ = 0.05956	P_2b_ = 0.16573	171 K**, 181 Q**,
Group 2c	ω_0(b)_ = 0.06509	ω_1(b)_ = 1.00000	ω_2a(b)_ = 0.06509	ω_2b(b)_ = 1.00000	192S**, 210 A**,
	ω_0(f)_ = 0.06509	ω_1(f)_ = 1.00000	ω_2a(f)_ = 5.55679	ω_2b(f)_ = 5.55679	237 S**, 243 I**
	P_0_ = 0.55167	P_1_ = 0.13832	P_2a_ = 0.24786	P_2b_ = 0.06215	25 N**, 26 I**, 34 C**, 79 S**,
Group 2d	ω_0(b)_ = 0.08684	ω_1(b)_ = 1.00000	ω_2a(b)_ = 0.08684	ω_2b(b)_ = 1.00000	148 T**, 208 G**, 214 D**,
	ω_0(f)_ = 0.08684	ω_1(f)_ = 1.00000	ω_2a(f)_ = 214.85997	ω_2b(f)_ = 214.85997	269 H**, 363 C**, 395 N**
	P_0_ = 0.27330	P_1_ = 0.60581	P_2a_ = 0.03758	P_2b_ = 0.08331	
Group 2e	ω_0(b)_ = 0.08686	ω_1(b)_ = 1.00000	ω_2a(b)_ = 0.08686	ω_2b(b)_ = 1.00000	196 E*
	ω_0(f)_ = 0.08686	ω_1(f)_ = 1.00000	ω_2a(f)_ = 44.60691	ω_2b(f)_ = 44.60691	
	P_0_ = 0.42970	P_1_ = 0.32076	P_2a_ = 0.14288	P_2b_ = 0.10666	
Group 3	ω_0(b)_ = 0.07702	ω_1(b)_ = 1.00000	ω_2a(b)_ = 0.07702	ω_2b(b)_ = 1.00000	107 F*
	ω_0(f)_ = 0.07702	ω_1(f)_ = 1.00000	ω_2a(f)_ = 999.00000	ω_2b(f)_ = 999.00000	

Note: *p < 0.05 and **p < 0.01 (x^2^ test).

Site class: The sites in the sequence evolve according to the same process, the transition probability matrix is calculated only once for all sites for each branch.

b: Background ω.

f: Foreground ω.

Positive delection sites: The number of amino acid sites estimated to have undergone positive selection.

BEB: Bayes Empirical Bayes.

### Functional divergence analysis (FDA) of SmWRKY proteins

Using DIVERGE 2.0 that evaluates shifted evolutionary rate and altered amino acid property after gene duplication [74,75], we carried out posterior analysis for estimation of Type-I and Type-II functional divergence between SmWRKY clusters. The estimation was based on the WRKY protein neighbor-joining tree consisting of three major groups (group 1, group 2a–e, and group 3) (Figure 4). Comparison among SmWRKY subgroups showed that all of the coefficients for the type I functional divergence (*θ*_I_) were greater than zero (Additional file 3: Table S3). The *θ*_I_ values of eight group pairs, including 1/2e, 1/3, 2a + b/2d, 2a + b/2e, 2a + b/3, 2c/2e, 2c/3 and 2d/3, were ranged from 0.219 to 0.772 and were statistically significant (*P* < 0.01) (Additional file 3: Table S3). It indicates that significant site-specific changes may have happened at certain amino acid sites between these group pairs, leading to a subgroup-specific functional evolution after their diversification.

**Figure 6:**
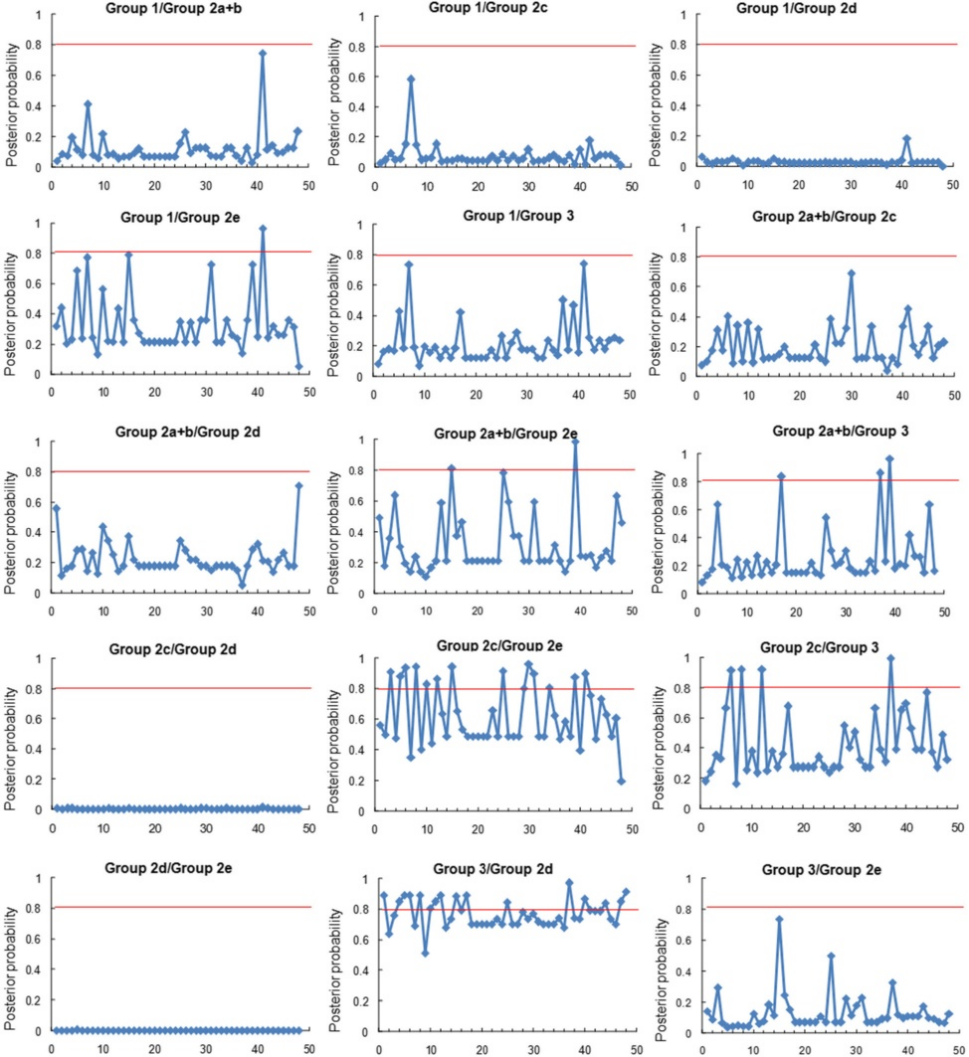
**Site-specific prediction for type-I functional divergence between groups of SmWRKYs.** The X-axis represents locations of sites. The Y-axis represents the probability of each group. The red line indicates cutoff = 0.80.

**Figure 7:**
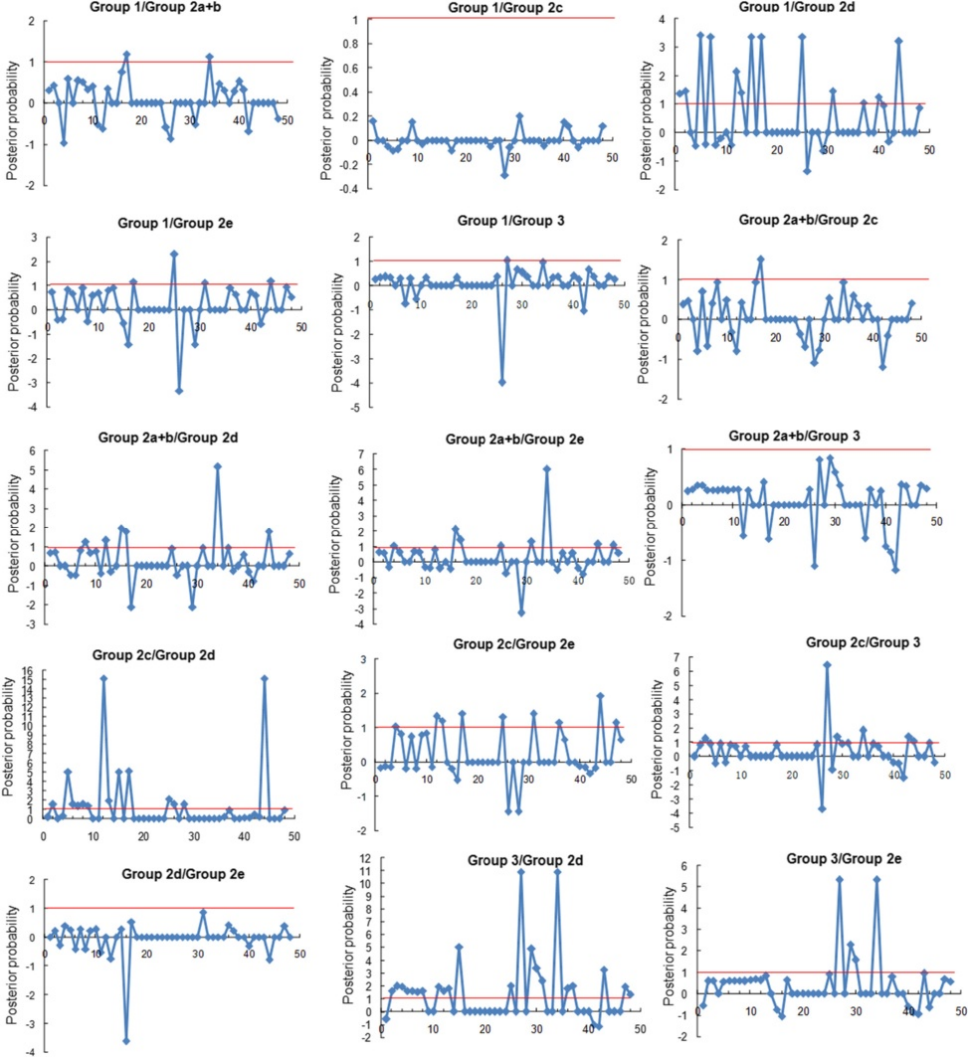
**Site-specific profile for predicting critical amino acid residues responsible for the type-II functional divergence between groups of SmWRKYs.** The X-axis represents locations of sites. The Y-axis represents the probability of each group. The red line indicates cutoff = 1.0.

Type II functional divergence (*θ*_II_) values in six group pairs, including 1/2c, 1/2d, 2a + b/2d, 2c/2d and 2d/3, were also greater than zero and ranged from 0.017 to 0.234 (Additional file 4: Table S4). It indicates a radical shift in amino acid properties. In order to extensively reduce positive false, *Q*_*k*_ > 0.8 and 1.0 were empirically used as cutoff in the identification of the Type-I and Type-II functional divergence-related residues between gene groups, respectively (Figures 6 and 7). Detailed analysis showed that the number and the distribution of predicted residues for functional divergence in group pairs were different (Additional file 3: Table S3 and Additional file 4: Table S4) and the residues predominantly existed in the WRKY domain. It suggests that these residues probably play important roles in functional divergence of WRKYs during the evolutionary process.

### Tissue-specific expression of *SmWRKYs*

**Figure 8:**
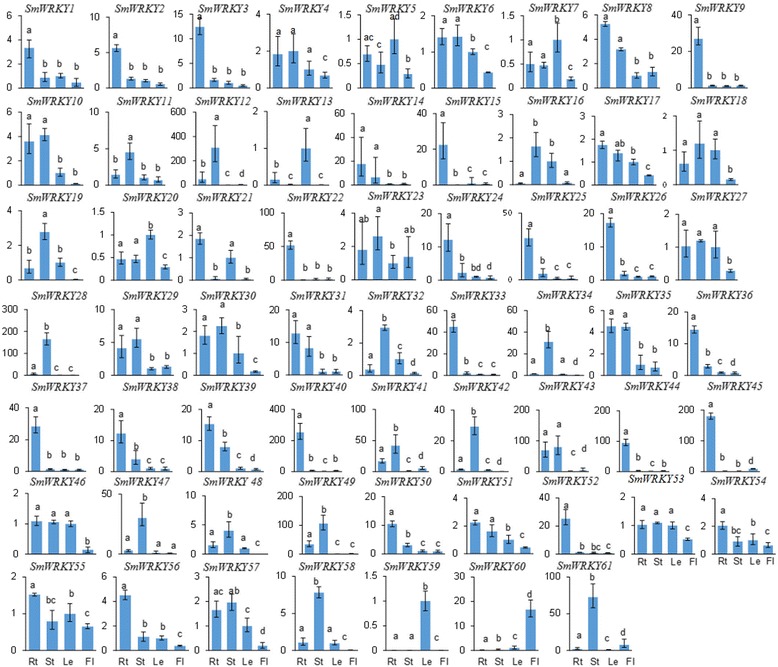
**Expression patterns of** ***SmWRKY*** **genes in roots (Rt), stems (St), leaves (Le) and flowers (Fl) of 2-year-old, field-grown** ***S. miltiorrhiza*** **Bunge (line 993).** The expression level of *SmWRKYs* was analyzed by the quantitative RT-PCR method. Y-axis indicates relative expression levels. X-axis indicates different tissues. *SmUBQ10* was used as the reference gene. Transcript levels in leaves were arbitrarily set to 1 and the levels in other tissues were given relative to this. Error bars represent standard deviations of mean value from three biological and three technical replicates. ANOVA (analysis of variance) was calculated using SPSS. *P* < 0.05 was considered statistically significant.

It has been shown that a number of WRKY proteins are involved in plant developmental processes [54,76,77]. In order to preliminarily understand the roles of *WRKYs* in *S. miltiorrhiza* development, we analyzed the expression of *SmWRKYs* in roots, stems, leaves and flowers of *S. miltiorrhiza* plants. All of the 61 *SmWRKYs* identified were expressed in at least a tissue analyzed and exhibited differential expression patterns (Figure 8). Of the 61 *SmWRKYs*, 22 (36.1%) showed predominant expression in roots, 13 (21.3%) in stems, 4 (6.6%) in leaves and 1 (1.6%) in flowers. The other 21 (34.4%) were mainly expressed in at least two tissues analyzed, indicating these genes are likely to play a more ubiquitous role in *S. miltiorrhiza*. Furthermore, some *SmWRKYs* in a group shared similar expression patterns, while the others were not. For example, *SmWRKY2*, *SmWRKY24*, *SmWRKY39*, *SmWRKY54* and *SmWRKY55*, belonging to group 1, were predominantly expressed in roots, while the other group 1 members, such as *SmWRKY42*, *SmWRKY13* and *SmWRKY60*, were mainly expressed in stems, leaves and flowers, respectively (Figure 8). It suggests that *SmWRKYs* belonging to a group do not necessarily indicate their functions in the same tissues. However, it has been shown that the tissues-specific expression patterns appear to be consistent with their role in the tissues. For example, VvWRKY01, belonging to group 2c, is involved in the regulation of lignin biosynthesis [78]. Over-expression of VvWRKY01 in tobacco resulted in the alteration of expression patterns of genes involved in lignin biosynthesis pathway [78]. Similarly, *SmWRKY12*, *SmWRKY19* and *SmWRKY47* in group 2c were predominantly expressed in stems (Figure 8). It indicates the putative roles of these *SmWRKYs* in the regulation of lignin biosynthesis in *S. miltiorrhiza. AtWRKY6*, a member of group 2b, and *AtWRKY53* and *AtWRKY70*, two *AtWRKYs* in group 3, are an important regulator in senescent leaves [55,76,79-81]. Of them, *AtWRKY6* acts in the upstream of SIRK during leaf senescence [55]. *SmWRKY59* belonging to the same WRKY group of *AtWRKY6* and *SmWRKY7* included in group 3 could be regulators of leaf senescence in *S. miltiorrhiza*, since both of them showed predominant expression in leaves (Figure 8). It has been shown that *AtWRKY44* included in group 1 is involved in trichome differentiation [54]. Several *SmWRKYs* in group 1, such as *SmWRKY2*, *SmWRKY39*, *SmWRKY24*, *SmWRKY54* and *SmWRKY55*, were highly expressed in roots with abundant root hairs, and *SmWRKY13* belonging to the same group was highly expressed in leaves with abundant of trichomes (Figure 8). It implies that these *SmWRKY* may be associated with trichome development in *S. miltiorrhiza*.

### Methyl jasmonate (MeJA)-responsive *SmWRKYs*

**Figure 9:**
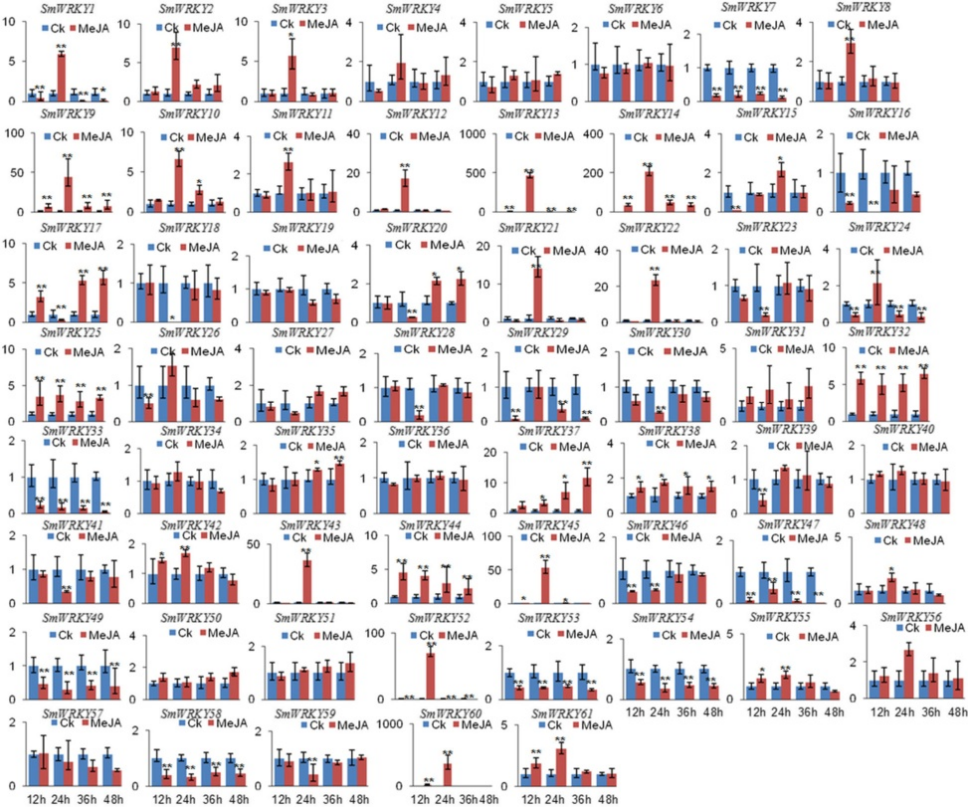
**Quantitative RT-PCR analysis of** ***SmWRKY*** **gene expression in** ***S. miltiorrhiza*** **roots treated with MeJA.** Fold changes of *SmWRKYs* in roots of *S. miltiorrhiza* plantlets treated with MeJA for 12, 24, 36 and 48 h are shown. *SmUBQ10* was used as the reference gene. The level of transcripts in roots treated with carrier solution (CK) was arbitrarily set to 1 and the levels in roots treated with MeJA were given relative to this. Mean values and SDs were obtained from three biological and three technical replicates. Y-axis indicates relative expression levels. X-axis indicates different time-points of MeJA treatment. ANOVA (analysis of variance) was calculated using SPSS. *P* < 0.05 (*) and *P* < 0.01(**) were considered statistically significant and extremely significant, respectively.

MeJA is a key signaling molecule involved in plant response to stress and in regulating secondary metabolite production in many plant species, including *S. miltiorrhiza* [3,14]. More than 50% (39 of 74) of *AtWRKYs* were MeJA-responsive in *Arabidopsis* [82]. More than 1/3 of *CrWRKY* were regulated by MeJA in hairy roots of *Catharanthus roseus* [83]. Additionally, *CaWRKY27* in *Capsicum annuum* [84], *GbWRKY1* in *Gossypium barbadense* [85] and *GhWRKY3* in *Gossypium hirsutum* [86] were also regulated by MeJA. In order to test whether *WRKYs* were responsive to MeJA treatment in *S. miltiorrhiza*, the expression level of *SmWRKYs* in roots of plantlets treated with MeJA was analyzed using the quantitative RT-PCR method. MeJA treatment showed a wide variety of *SmWRKY* gene expression profiles (Figure 9). Significant expression level changes were observed for 49 *SmWRKYs*, of which 26 were up-regulated, 18 were down-regulated, while the other 5, including *SmWRKY1*, *SmWRKY15*, *SmWRKY17*, *SmWRKY20* and *SmWRKY24*, were either up-regulated or down-regulated at different time-points of treatment (Figure 9). It suggests that about 80% of the *SmWRKYs* analyzed are MeJA-responsive. Examination of the number of *SmWRKYs* with significant expression level changed at different time-points of treatment showed that the expression of 28, 43, 25 and 23 *SmWRKYs* was changed after MeJA treatment for 12, 24, 36, and 48 hours, respectively (Figure 9). It suggests that the majority of *SmWRKYs* have altered expression levels at the time-point of 24 h-treatment. The number of up-regulated *SmWRKYs* was 13, 26, 15, and 13 at the time-point of 12, 24, 36, and 48 hours, respectively, while that of down-regulated was 15, 17, 10, and 10, respectively. Additionally, 8 *SmWRKYs*, including *SmWRKY9*, *SmWRKY13*, *SmWRKY14*, *SmWRKY25*, *SmWRKY32*, *SmWRKY38*, *SmWRKY44* and *SmWRKY52*, were significantly up-regulated at all time-points of MeJA treatment, while 7, including *SmWRKY7*, *SmWRKY33*, *SmWRKY47*, *SmWRKY49*, *SmWRKY53*, *SmWRKY54* and *SmWRKY58*, were down-regulated (Figure 9). It suggests that the number of up-regulated *SmWRKYs* is slightly more than down-regulated.

### Yeast extract and Ag^+^-responsive *SmWRKYs*

In order to further investigate the roles of *SmWRKYs* in *S. miltiorrhiza*, transcriptome-wide analysis of *SmWRKY* expression in response to yeast extract and Ag^+^ treatment was performed. RNA-seq data of *S. miltiorrhiza* hairy roots treated with or without yeast extract (100 μg/ml) and Ag^+^ (30 μM) were downloaded from GenBank under the accession number SRR924662 [12]. RNA-seq reads from non-treated (0 hpi) and treated for 12 h (12 hpi), 24 h (24 hpi) and 36 h (36 hpi) were mapped to *SmWRKYs* using the SOAP2 software [87]. The log-2-transformed RPKM (RNA-seq reads mapped to a *SmWRKY* per total million reads from a treatment per kilobases of the *SmWRKY* length) value of *SmWRKYs* varied between −3.04 and 8.38 (Additional file 5: Table S5). Using a cutoff of RPKM value >2.0, a total of 49 *SmWRKYs* were found to be expressed in hairy roots. Fisher’s exact test showed that 42 of the 49 *SmWRKYs* were differentially expressed (Additional file 5: Table S5). It includes 17 significantly up-regulated, 19 significantly down-regulated and 6 significantly up- or down-regulated at different time-points, suggesting the majority of the identified *SmWRKYs* were responsive to yeast extract and Ag^+^ treatment.

### *SmWRKY* candidate*s* potentially involved in tanshinone biosynthesis

Terpenoids are plant secondary metabolites with significant physiological and ecological functions and great economic values, and a class of terpenoids, known as tanshinones, is the main bioactive compounds in *S. miltiorrhiza*. Increasing evidence demonstrates the importance of *WRKY* genes in the biosynthesis of secondary metabolites, such as terpenoid indole alkaloid in *Catharanthus roseus* [83]. Additionally, it has been shown that *Gossypium arboretum GaWRKY1*, which belongs to group 2a, participates in sesquiterpene biosynthesis in cotton by controlling (+)-δ-cadinene synthase (CAD1) activity [88]. *PqWRKY1*, a member of group 2d, responds to MeJA treatment and is a positive regulator of osmotic stress response and triterpene ginsenoside biosynthesis in *Panax quinquefolius* [89]. *AaWRKY1* and *CrWRKY*, belonging to group 3, are the other two terpenoid biosynthesis-related *WRKYs. Artemisia annua AaWRKY1* was highly expressed in glandular secretory trichomes (GSTs), where the sesquiterpene artemisinin was synthesized [90]. *AaWRKY1* might be strongly induced by MeJA and could bind to the W-box in the promoter of *ADS* gene encoding amorpha-4, 11-diene synthase, a key enzyme in the artemisinin biosynthesis pathway [90]. *CrWRKY1* was preferentially expressed in roots of *C. roseus* and also induced by MeJA [91]. It controlled terpenoid indole alkaloid biosynthesis through positive regulation of *DXS* and *SLS* genes involved in the terpenoid pathway and *AS* and *TDC* genes involved in the indole pathway [91]. Thus, *SmWRKYs* included in group 2a, 2d and 3 probably have an evolutionarily conserved role in regulating terpenoid biosynthesis in *S. miltiorrhiza*.

Terpenoid tanshinones have been mainly produced and accumulated in roots of field-grown *S. miltiorrhiza* during the fast growing period from June to September [92-94]. The process of tanshinone production may be stimulated by MeJA, yeast extract and Ag^+^ [10,12,95]. Among the 61 identified *SmWRKYs*, sixteen showed similar responses to the MeJA treatment and the yeast extract and Ag^+^ treatment (Figure 9, Additional file 5: Table S5). *SmWRKY2*, *SmWRKY3*, *SmWRKY9*, *SmWRKY11*, *SmWRKY25*, *SmWRKY32*, *SmWRKY37*, *SmWRKY43* and *SmWRKY52* were up-regulated by both the MeJA treatment and the yeast extract and Ag^+^ treatment, while *SmWRKY23*, *SmWRKY26*, *SmWRKY33*, *SmWRKY41*, *SmWRKY47*, *SmWRKY53* and *SmWRKY59* were down-regulated (Figure 9, Additional file 5: Table S5). Among the sixteen *SmWRKYs*, eight, including six up-regulated (*SmWRKY2*, *SmWRKY3*, *SmWRKY9*, *SmWRKY25*, *SmWRKY37* and *SmWRKY52*) and two down-regulated (*SmWRKY26* and *SmWRKY33*), were predominantly expressed in roots of field-grown *S. miltiorrhiza* in August (Figure 8), suggesting their specific roles in roots. *SmWRKY2*, *SmWRKY3*, *SmWRKY9* and *SmWRKY26* are members of groups 1, 2d, 2a and 2b, respectively, while *SmWRKY25*, *SmWRKY33*, *SmWRKY37* and *SmWRKY52* are members of group 2c (Figures 1 and 4). Thus, *SmWRKY3* and *SmWRKY9* are two *SmWRKYs* (1) with similar responses to the MeJA treatment and the yeast extract and Ag^+^ treatment, (2) having root-specific expression, and (3) belonging to group 2a, 2d or 3 with members probably playing an evolutionarily conserved role in regulating terpenoid biosynthesis. It implicates that *SmWRKY3* and *SmWRKY9* are very likely to be activators in tanshinone production. Notably, we may not exclude the possibility that some other *SmWRKYs* are also involved in tanshinone biosynthesis based on the data currently available. Further analysis of transgenic *S. miltiorrhiza* plants with over-expressed or silenced *SmWRKYs* may help to elucidate their function.

### Divergence of paralogous *SmWRKY* genes

Gene duplication is an important event for gene family expansion and functional diversity during evolution [65,96,97]. A total of 42 (68.85% of 61) *SmWRKY* genes appear to be duplicated (Additional file 5: Table S5). In order to preliminarily reveal the mechanism of functional diversity (nonfunctionalization, subfunctionalization and neofunctionalization [98]) of these genes after duplication, the synonymous (Ks) and non-synonymous (Ka) substitution rate were calculated using the CDS of paralogous *SmWRKY* genes (Additional file 6: Table S6). The Ka/Ks ratios for all of the 21 paralogous *SmWRKY* gene pairs were less than one with 5 pairs even close to zero. It suggests that the *WRKY* genes from *S. miltiorrhiza* have experienced strong purifying selection pressure. Some closely related gene pairs displayed different expression patterns, indicating functional divergences occurred. For example, *SmWRKY13* was expressed dominantly in leaves, whereas the other member of the *SmWRKY13/SmWRKY31* gene pair, *SmWRKY31*, was expressed mainly in roots and stems (Figure 8). In addition, *SmWRKY13*, but not *SmWRKY31*, was significantly up-regulated by MeJA (Figure 9). Expression patterns of other paralogous genes, such as *SmWRKY23/49*, *SmWRKY30/35*, *SmWRKY41/55*, *SmWRKY42/53*, and *SmWRKY43/SmWRKY52*, were also different (Figures 8 and 9). It indicates that many *SmWRKY* gene pairs are divergent under the purifying pressure [99].

## Conclusions

In this study, we cloned a total of 61 *SmWRKY* genes. The cloned genes and the deduced proteins were characterized through a comprehensive approach, including phylogenetic tree construction, WRKY domain characterization, conserved motif identification, selective constraint analysis, functional divergence analysis, and expression profiling. We showed that many *SmWRKY*s and *AtWRKYs* were evolutionarily conserved. The WRKY domains could be divided into 3 groups (1, 2 and 3) and 8 subgroups (1 N, 1C, 2a, 2b, 2c, 2d, 2e and 3). Each group of WRKY domains contains characteristic conserved sequences. Additionally, sequence outside the WRKY domain might contribute to the difference between the phylogenetic tree constructed with the WRKY domains and that constructed with the whole WRKY proteins. A total of 20 conserved motifs were identified, of which group-specific motifs might attribute to functional divergence of WRKYs. We identified 17 pairs of orthologous *SmWRKY* and *AtWRKY* genes and 21 pairs of paralogous *SmWRKY* genes. Selective constraint analysis and functional divergence analysis showed that the *SmWRKY* subgroup genes have experienced strong positive selection and diverged in function. Gene expression profiles suggest that the majority of 61 *SmWRKY* genes are tissue-specific and MeJA- and yeast extract and Ag^+^-responsive. These results provide insights into functional conservation and diversification of *SmWRKYs* and are useful for further investigating *SmWRKY* functions in *S. miltiorrhiza* development and defense response.

## Methods

### Database search and sequence annotation

The nucleotide sequences and amino acids of 72 *AtWRKY* genes were obtained from the *Arabidopsis* Information Resource (TAIR; http://www.Arabidopsis.org/). *S. miltiorrhiza WRKY* (*SmWRKY*) genes were predicted by tBLASTn [100] search of *AtWRKY* [101] homologs against the current *S. miltiorrhiza* genome assembly, which covers about 92% of the entire genome and 96% of the protein-coding genes [18]. An e-value cut-off of 1e-10 was applied to the homologue recognition. The retrieved sequences were used for gene model prediction on the GENSCAN web server (http://genes.mit.edu/GENSCAN.html). Full-length CDSs of *SmWRKYs* were amplified by reverse transcription-PCR using the primers listed in Additional file 2: Table S2. PCR products were gel-purified, cloned, and then sequenced. The theoretical isoelectric point (p*I*) and molecular weight (Mw) were predicted using the Compute p*I*/Mw tool on the ExPASy server (http://web.expasy.org/compute_pi/) [102].

### Multiple sequence alignment, phylogenetic analysis and motif detection

*PpWRKY* genes were obtained from *Physcomitrella patens* v3.1 (http://phytozome.jgi.doe.gov/pz/portal.html#!info?alias=Org_Ppatens_er). Human FLYWCH CRAa (EAW85450) and GCMa (BAA13651) were obtained from NCBI (http://www.ncbi.nlm.nih.gov/protein/). Multiple sequence alignment of the WRKY domain from 61 *S. miltiorrhiza* SmWRKYs and 72 *Arabidopsis* AtWRKYs was performed using CLUSTALW with BOXSHADE (http://bioweb.pasteur.fr) [103]. Phylogenetic trees were constructed using MEGA 5.0 with the neighbor-joining method [104]. Bootstrap test was replicated 1000 times. Motifs were detected using MEME 5.0 [105].

### Plant materials and MeJA treatment

Roots, stems, leaves and flowers from 2-year-old, field-grown *S. miltiorrhiza* Bunge (line 993) plants were collected in August, 2012 and stored in liquid nitrogen until use. Plantlets cultivated *in vitro* were grown at 25°C with a photoperiod of 16 h light and 8 h dark for six weeks and treated with 200 μM methyl jasmonate (MeJA) for 12, 24, 36 and 48 h as described previously [3,63]. Plantlets treated with carrier solution were used as controls. Roots of plantlets with or without MeJA treatment were collected and stored in liquid nitrogen until use. Three independent biological replicates were carried out for each experiment.

### RNA extraction and quantitative real-time reverse transcription-PCR (qRT-PCR)

Total RNA was extracted from plant tissues using the Quick RNA Isolation Kit (Huayueyang, China). RNA integrity was analyzed on a 1.2% agarose gel. RNA quantity was determined using a NanoDrop 2000C Spectrophotometer (Thermo Scientific, USA). cDNA synthesis was carried out using Superscript III Reverse Transcriptase (TaKaRa, China). qRT-PCRs were performed using the SYBR premix Ex Taq™ kit (TaKaRa, China) and carried out in triplicate for each tissue sample. Gene-specific primers (Additional file 7: Table S7) were designed using Primer Premier 5.0. The length of amplicons is between 80 bp and 250 bp. *SmUBQ10* was selected as a reference gene as described previously [3]. Three independent biological replicates were performed. Statistical analysis was carried out as described [20]. Briefly, standardization of gene expression data was performed from three biological replicates as described [106]. 2-ΔΔCq was used to achieve results for relative quantification. For statistical analysis, ANOVA (analysis of variance) was calculated using SPSS (Version 19.0, IBM, USA).

### Analysis of *SmWRKY* expression in response to yeast extract and Ag^+^ treatment

RNA-seq data for *S. miltiorrhiza* hairy roots treated with yeast extract (100 μg/ml) and Ag^+^ (30 μM) were downloaded from GenBank under the accession number SRR924662 [12]. RNA-seq reads from non-treated (0 hpi) and treated for 12 h (12 hpi), 24 h (24 hpi) and 36 h (36 hpi) were mapped to *SmWRKYs* using the SOAP2 software [87] and analyzed as described previously [107]. The parameter v cutoff of 3 and parameter r cutoff of 2 were applied. *SmWRKYs* with the RPKM value greater than 2 were analyzed for differential expression using Fisher’s exact test. *P* < 0.05 was considered as differentially expressed.

### Ka and Ks calculation

Paralogous *SmWRKY* genes were inferred from phylogenetic analysis. Non-synonymous (Ka) and synonymous (Ks) substitution of each paralogous gene pair were also determined by PAL2NAL program (http://www.bork.embl.de/pal2nal/) [108], which is based on the codon model program in PAML [68].

### Tests of positive selection

To determine whether the WRKY gene family exhibited evidence of positive selection under the site model and branch-site model [71], we applied the codeml program in PAML v4.8 to test the hypothesis of positive selection. An unrooted phylogenetic tree of SmWRKYs was reconstructed using the maximum likelihood method. In the site model, M0 (one ratio), M1a (neutral), M2a (selection), M3 (discrete), M7 (beta) and M8 (beta + ω > 1) were applied to the alignments, and we detected variation in the ω parameter among sites using the LRT for M1a vs. M2 a, M0 vs. M3 and M7 vs. M8. Branch-site model [72] was used to compare the non-synonymous/synonymous substitution rate ratio (Ka/Ks) between clades or sequences. The ratio of nonsynonymous-to-synonymous for each branch under model A was calculated. Posterior probabilities (Qks) were calculated using the BEB method [68].

### Estimation of functional divergence

The software DIVERGE2 was used to detect the functional divergence among members of SmWRKY subgroups [74]. The method is based on maximum likelihood procedures to estimate significant changes in the site-specific shift. The coefficients of Type-I and Type-II functional divergence (θ_I_ and θ_II_) between two clusters were calculated. The coefficients of Type-I and Type-II functional divergence (θ_I_ and θ_II_) greater than 0 indicates that site specific altered selective constraints or a radical shift of amino acid physiochemical property occurred after gene duplication and/or speciation [74]. Large posterior probability (Qk) indicates a high possibility that the functional constraint (or the evolutionary rate) and/or the radical change in the amino acid property of a site is different between two clusters [74].

### Availability of supporting data

*SmWRKY* sequences supporting the results of this article are available in GenBank under accession numbers KM823124–KM823184. The other supporting data are included within the article and its additional files.

## Additional files

Additional file 1: Table S1. Sequence features of WRKYs in *A. thaliana*. Some sequence features of WRKYs in *A. thaliana* are shown. Additional file 2: Table S2. Primers used for cloning of *SmWRKY* CDSs. Complete set of primers used for amplification of *SmWRKY* CDSs. Additional file 3: Table S3. Maximum likelihood estimation of the coefficient of Type-I functional divergence (θ) from pairwise comparisons between WRKY groups. The coefficient of Type-I between WRKY groups is shown. Additional file 4: Table S4. Estimation of the coefficient of Type-II functional divergence (θ) from pairwise comparisons between WRKY groups. The coefficient of Type-II between WRKY groups is shown. Additional file 5: Table S5. Log-2 transformed RPKM value for *SmWRKYs* in hairy roots treated with or without yeast extract and Ag^+^. The transformed RPKM value and differential expression of *SmWRKYs* are shown. Additional file 6: Table S6.
*Ka*/*Ks* and divergence analysis of paralogous *SmWRKY* genes. *Ka*, *Ks* and *Ka*/*Ks* are shown. Additional file 7: Table S7. Primers used for qRT-PCR analysis of *SmWRKY* genes. Complete set of primers used for qRT-PCR of *SmWRKY* genes.

## Abbreviations

AS: Anthranilate synthase
BEB: Bayes empirical bayes
CAD1: (+)-δ-cadinene synthase
CDS: Coding sequence
Ka: Non-synonymous
Ks: Synonymous
*DXS*: 1-deoxy-D-xylulose 5-phosphate synthase
GST: Glandular secretory trichome
LRT: Likelihood ratio test
MeJA: Methyl jasmonate
Mw: Molecular weight
p*I*: Isoelectric points
SLS: Secologanin synthase
TCM: Traditional Chinese medicine
TDC: Tryptophan decarboxylase
