# Supplementary material for: Molecular cloning and expression analysis of WRKY transcription factor genes in Salvia miltiorrhiza
Source: BMC Genomics. 2015 Mar 17;16(1):200. doi: 10.1186/s12864-015-1411-x (PMC4371873; doi:10.1186/s12864-015-1411-x)
Supplement: Additional file 3: Table S3. — Maximum likelihood estimation of the coefficient of Type-I functional divergence (θ) from pairwise comparisons between WRKY groups. The coefficient of Type-I between WRKY groups is shown. [file 12864_2015_1411_MOESM3_ESM.doc]

**Table S3.** Maximum likelihood estimates of the coefficient of Type-I functional divergence (θ) from pairwise comparisons between WRKY groups

| Group 1 | Group 2 | θ_I_ ± S.E. | θ _LRT_ | *Q_k_*>0.8 | *Q_k_*>0.9 | P |
| --- | --- | --- | --- | --- | --- | --- |
| Group 1 | Group 2a+b | 0.121 ± 0.105 | 1.320 | 0 | 0 |  |
| Group 1 | Group 2c | 0.075 ± 0.102 | 0.546 | 0 | 0 |  |
| Group 1 | Group 2d | 0.031 ± 0.170 | 0.032 | 0 | 0 |  |
| Group 1 | Group 2e | 0.331 ± 0.115 | 8.249 | 1 | 1 | P<0.01 |
| Group 1 | Group 3 | 0.220 ± 0.140 | 2.459 | 0 | 0 | P<0.01 |
| Group 2a+b | Group 2c | 0.199 ± 0.164 | 1.472 | 0 | 0 |  |
| Group 2a+b | Group 2d | 0.226 ± 0.192 | 1.395 | 0 | 0 | P<0.01 |
| Group 2a+b | Group 2e | 0.322 ± 0.102 | 9.900 | 1 | 1 | P<0.01 |
| Group 2a+b | Group 3 | 0.262 ± 0.089 | 8.716 | 3 | 1 | P<0.01 |
| Group 2c | Group 2d | 0.003 ± 0.076 | 0 | 0 | 0 |  |
| Group 2c | Group 2e | 0.624 ± 0.133 | 21.861 | 14 | 6 | P<0.01 |
| Group 2c | Group 3 | 0.422 ± 0.102 | 17.187 | 4 | 4 | P<0.01 |
| Group 2d | Group 2e | 0.001 ± 0.022 | 0 | 0 | 0 |  |
| Group 2d | Group 3 | 0.772 ± 0.248 | 9.652 | 16 | 2 | P<0.01 |
| Group 2e | Group 3 | 0.131 ± 0.107 | 1.505 | 0 | 0 |  |

Note: *x*^2^ test was introduced to examined the data with statistically significant difference.

Posterior probability (PP) : the site-specific Type-I functional divergence.
