# Supplementary material for: Molecular cloning and expression analysis of WRKY transcription factor genes in Salvia miltiorrhiza
Source: BMC Genomics. 2015 Mar 17;16(1):200. doi: 10.1186/s12864-015-1411-x (PMC4371873; doi:10.1186/s12864-015-1411-x)
Supplement: Additional file 4: Table S4. — Estimation of the coefficient of Type-II functional divergence (θ) from pairwise comparisons between WRKY groups. The coefficient of Type-II between WRKY groups is shown. [file 12864_2015_1411_MOESM4_ESM.doc]

**Table S4:** Estimates of the coefficient of Type-II functional divergence (θ)

| Group 1 | Group 2 | θ_II_ ± S.E. | *Q_k_*>1.0 |
| --- | --- | --- | --- |
| Group 1 | Group 2a+b | -0.156 ± 0.306 | 2 |
| Group 1 | Group 2c | 0.017 ± 0.260 | 0 |
| Group 1 | Group 2d | 0.121 ± 0.233 | 13 |
| Group 1 | Group 2e | -0.072± 0.284 | 4 |
| Group 1 | Group 3 | -0.334 ± 0.400 | 1 |
| Group 2a+b | Group 2c | -0.083 ± 0.256 | 1 |
| Group 2a+b | Group 2d | 0.017 ± 0.200 | 6 |
| Group 2a+b | Group 2e | -0.032 ± 0.244 | 8 |
| Group 2a+b | Group 3 | -0.330 ± 0.352 | 0 |
| Group 2c | Group 2d | 0.234 ± 0.172 | 14 |
| Group 2c | Group 2e | 0.104 ± 0.224 | 9 |
| Group 2c | Group 3 | -0.051 ± 0.296 | 6 |
| Group 2d | Group 2e | -0.124 ± 0.200 | 0 |
| Group 2d | Group 3 | 0.070 ± 0.262 | 22 |
| Group 2e | Group 3 | -0.142 ± 0.321 | 4 |
